# Current Treatment Options for Children with Functional Constipation—What Is in the Pipeline?

**DOI:** 10.3390/children12070857

**Published:** 2025-06-28

**Authors:** Charlotte A. L. Jonker, Tirza M. van Os, Ramon R. Gorter, Marc A. Levitt, Marc A. Benninga

**Affiliations:** 1 Department of Pediatric Gastroenterology and Nutrition, Emma Children’s Hospital, Amsterdam UMC, University of Amsterdam, 1105 AZ Amsterdam, The Netherlands; cjonker@childrensnational.org (C.A.L.J.); m.a.benninga@amsterdamumc.nl (M.A.B.); 2 Department of Pediatric Surgery, Emma Children’s Hospital, Amsterdam UMC, University of Amsterdam, 1105 AZ Amsterdam, The Netherlands; rr.gorter@amsterdamumc.nl; 3 Division of Colorectal and Pelvic Reconstruction, Children’s National Hospital, Washington, DC 20010, USA; mlevitt@childrensnational.org; 4 Gastroenterology and Hepatology, Amsterdam Gastroenterology Endocrinology Metabolism, Amsterdam UMC, University of Amsterdam, Meibergdreef 9, 1105 AZ Amsterdam, The Netherlands; 5 Amsterdam Reproduction and Development Research Institute, Amsterdam UMC, University of Amsterdam, Meibergdreef 9, 1105 AZ Amsterdam, The Netherlands

**Keywords:** functional constipation, pediatrics, surgery, new pharmacological approaches, neurostimulation

## Abstract

In this review, we summarize current insights into the treatment of functional constipation (FC) in children. Constipation is a global issue in the pediatric population, with a prevalence of approximately 9.5%. Initial management involves a combination of non-pharmacological and pharmacological interventions. However, a significant number of children continue to experience therapy-resistant FC despite optimal non-pharmacological and pharmacological treatments. While studies on novel pharmacological options in children are limited, adult trials have shown promising results. New agents such as lubiprostone, prucalopride, linaclotide, and plecanatide have demonstrated improved outcomes compared to placebo or conventional therapies, particularly in increasing spontaneous bowel movements. Neurostimulation presents an additional treatment modality. Posterior tibial nerve stimulation appears to be a promising new option, offering high treatment satisfaction and a favorable safety profile with a low rate of severe adverse events. For children who do not respond to optimal conservative therapy, the impact on quality of life can be substantial. In such cases, surgical interventions may be considered, including intrasphincteric botulinum toxin injections, antegrade continence enema surgery, and, in severe cases, colonic resection or a diverting ostomy. The choice of surgical treatment remains a subject of ongoing debate. Therapy-resistant FC in children is a complex and impactful condition. An individualized, stepwise approach is essential, with surgical options such as colonic resection reserved as a last resort.

## 1. Introduction

Constipation in children is a global problem, with a pooled prevalence of 8.17% (95% CI: 6.33–10.22%) for children from 0 to 4 years old and 11.39% (95% CI: 9.34–14.11%) for children 4–18 years old [1,2]. Before a diagnosis of functional constipation (FC) can be established, organic causes must be excluded. It is essential to perform a thorough physical examination and, if necessary, to conduct additional diagnostic procedures, such as contrast enemas or obtaining anal/rectal biopsies. Possible missed diagnoses could be ultra short Hirschsprung disease (HD), a subtle recto-perineal fistula or a thickened internal anal sphincter [3,4,5]. Neurologic (e.g., spinal cord malformations, pseudo-obstruction syndrome), metabolic or endocrine disorders (e.g., coeliac disease, cystic fibrosis, hypothyroidism) could also cause constipation without clear physical malformations [6]. Otherwise, according to the pediatric Rome IV criteria, FC is diagnosed in children of all ages when two or more of the following symptoms are present for at least one month: (1) fewer than three bowel movements per week, (2) at least one episode of fecal incontinence (FI) per week, (3) a history of excessive stool retention, (4) a history of painful or hard bowel movements, (5) the presence of a large fecal mass in the rectum, and (6) large-diameter stools that may obstruct the toilet [7,8].

Initial treatment of FC typically involves a combination of non-pharmacological and pharmacological approaches [9]. In clinical practice, the most commonly used first-line osmotic laxatives are polyethylene glycol (PEG) and lactulose, while bisacodyl or senna are the stimulant laxatives of choice [10]. However, despite optimal conservative treatment, a significant group of children continue to experience constipation, which can negatively affect their quality of life (QoL). It has been reported that approximately 50% of children referred to a pediatric gastroenterologist remain symptomatic after five years, and around 20% continue to struggle with symptoms of constipation even after ten years despite intensive treatment [11]. In such cases, more invasive interventions, such as surgery, may be necessary.

Although there is no consensus definition in the terminology, and the maximal medical treatment and duration of medical intervention needed before concluding that the patient is medically unresponsive constipation varies amongst clinicians, for the purposes of this review, we will refer to medically unresponsive constipation as therapy-resistant constipation [12]. Conventional laxative and enema-based treatments often do not bring ultimate patient satisfaction and a decrease in symptoms, but novel therapy options are being developed rapidly. This review provides an update on both new pharmacological and surgical management options for children with FC.

## 2. New Medications

For adults with FC, more and more pharmacological options are available. When a successful response rate is achieved in the adult population, studies in the pediatric population are performed. An overview of the following four mentioned drugs can be found in Table 1.

### 2.1. Lubiprostone

Lubiprostone, difluoropentyl-2-hydroxy-6-oxooctahydrocyclopenta-heptanoic acid, activates the chloride channels 2 (ClC-2) located on the gastrointestinal apical cell membrane. When these channels are activated, fluid secretion will be established and softens the feces, which supports transit through the gastrointestinal tract [13].

Large randomized controlled trials (n = 237 and n = 1171, respectively) evaluating the effect of lubiprostone in adults with FC and/or IBS-C reported promising results with significantly more spontaneous bowel movements (SBMs) [14,15].

In the past five years, several studies have been performed to identify the efficacy and safety of lubiprostone in children with FC. A non-randomized open-label study evaluated the safety of lubiprostone in children fulfilling the Rome III criteria for FC at 6–17 years of age. Indeed, lubiprostone (12 mcg or 24 mcg orally) was found to be well tolerated and without significant adverse events (AEs) [16]. Treatment-emergent adverse events were mostly mild in intensity, with gastrointestinal disorders (diarrhea, vomiting) most frequently reported and similar to those reported in adult studies. Subsequently, a multicenter, randomized, double-blind, placebo-controlled study [17], in which 444 children, 6–17 years of age, and fulfilling the Rome III criteria for FC, used lubiprostone or placebo for a minimum of twelve weeks, showed no significant difference in SBMs between the two groups. Indeed, a systematic review evaluating the efficacy and safety of treatments used for intractable constipation in children confirmed that lubiprostone made little to no difference in treatment success (RR 1.29, 95% CI 0.87 to 1.92; low certainty evidence) [18,19]. Recently, a randomized controlled, single-blinded trial in 280 children with FC, 8–18 years of age, evaluated the efficacy of lubiprostone compared to one of the conventional therapies (bisacodyl, sodium picosulfate or lactulose). In contrast to the aforementioned study, results showed that the number of spontaneous bowel movements (SBMs) when using lubiprostone was significantly higher compared to conventional therapies. More research is needed to identify its usefulness since no clear efficacy has been revealed yet. Until then, lubiprostone will not be a standard laxative therapy for children with FC.

### 2.2. Linaclotide

Linaclotide is a guanylate cyclase-C (GC-C) receptor agonist. Activating the GC-C receptor leads to more intestinal fluids, softens feces, and speeds up the transit of stool [20].

In recent years, several studies in adults with FC have shown that linaclotide is an effective laxative treatment [21]. In 2021, a study reported on 93 children, 8–17 years of age, fulfilling the Rome IV criteria for irritable bowel syndrome with constipation (IBS-C} (n = 60) and FC (n = 33) [22]. Linaclotide significantly improved the amount of bowel movements in these children. However, AEs were reported in 35% of the population, and therefore, 20% stopped using linaclotide. The most common adverse events reported were diarrhea and flatulence.

A large study conducted in seven countries evaluated the difference in SBMs when using linaclotide (n = 166) compared to placebo (n = 164) in children, 6–17 years of age, fulfilling the Rome III criteria for FC [23]. Compared with placebo, patients treated with linaclotide showed significant improvement in SBM frequency and stool consistency (*p* < 0.001). Baseline SBM in the linaclotide group was 1.16 times a week, which increased to 3.41 SBMs a week. In the placebo group, baseline SBM was 1.28 a week compared to 2.29 a week after intervention. The most frequent treatment-related AE was diarrhea (linaclotide: six (4%) patients; placebo: two (1%) patients). To our knowledge, these are the only published studies with children with FC included. More prospective trials are needed, and over a longer period of time, to be able to draw any conclusions about linaclotide.

### 2.3. Prucalopride

Prucalopride is a selective, high-affinity 5-hydroxytryptamine-4 (5-HT-4) receptor agonist. It is a pro-kinetic drug which, in contrast to the other mentioned medicines, stimulates motility in the gastro-intestinal tract [24,25,26].

In adults with FC, studies have shown promising results with an increase in SBM and an acceptable number of adverse events when prucalopride is used for the treatment of FC [25,27]. The most common AEs were headache, nausea, abdominal pain and diarrhea. No significant differences were shown in the number of AEs in adults using prucalopride or placebo [27,28]. Also, most AEs were transient and mainly present within the first 24 h of treatment. To date, only a few studies have been performed to evaluate prucalopride for the treatment of FC in children. In 2014, a large (n = 213), double-blinded randomized controlled trial (RCT) compared prucalopride placebo in children, from 6 months to 18 years of age, fulfilling the Rome III criteria for FC [29]. In contrast to the results in adults, this study showed that there was no significant difference in meeting the endpoints (i.e., ≥3 SBMs a week and ≤1 episode of FI in two weeks) in using prucalopride compared to placebo in children with FC. Headache was the most reported AE. After day one, AEs were reported in 62.3% and 57.9% of the patients using prucalopride or the placebo, respectively.

A more recent study compared the use of conventional therapy with the use of conventional therapy (i.e., rectal disimpaction, bowel cleanout, osmotic or stimulative laxatives, pelvic floor physical therapy and modification in behavior) in combination with prucalopride [30]. The combination of both compounds was not superior to conventional therapy alone.

The difference in efficacy between adults and children can be explained by the different pathophysiology of FC between those two groups. In contrast to adults with constipation, the underlying mechanism of constipation in children is withholding behavior. Since prucalopride works as a stimulative laxative, the impact in adults is bigger since slow bowel transit is more often the cause of constipation compared to children [31]. Based on the current evidence, prucalopride cannot be recommended for routine use in children with FC.

### 2.4. Plecanatide

Plecanatide is a 16-amino-acid peptide analog of uroguanylin, an active stimulator of the guanylate cyclase-C receptors. It eventually leads to an increase in water secretion, which softens the stool [32].

In adults with FC, some studies evaluating the efficacy of plecanatide have been completed with potentially positive conclusions [33]. An RCT performed by Cash et al. included 646 patients with chronic idiopathic constipation and 527 patients with IBS-C [34]. There was a significant difference in response rate when using Plecanatide (3 mg or 6 mg) compared to placebo. Another study, including 1394 patients, also compared placebo with 2 doses (3 mg and 6 mg) of plecanatide in adults with FC. Results showed a significant difference in SBM using 3 mg or 6 mg plecanatide compared to placebo, and a low rate of adverse events [35].

To date, no clinical trials are published in which children with FC are included. Currently, there is one clinical trial (NCT03596905) underway, comparing plecanatide with placebo in children with IBS-C, 6–17 years of age. Another registered clinical trial in adolescents with FC plans to evaluate the efficacy and safety of plecanatide as a treatment option for FC (NCT03120520).

## 3. An Insight into Future Study Directions

Tenapanor (a sodium-hydrogen exchanger isoform 3 inhibitor) was recently added in the treatment of IBS-C in adults [36]. It significantly improves IBS-C-related symptoms (i.e., daily bowel movements and abdominal pain) compared with placebo [37]. AEs that were reported were mild and mainly diarrhea and flatulence. Another new option is mizagliflozin (a sodium-glucose cotransporter 1 inhibitor) [38]. The use of 5 mg or 10 mg showed significant improvement in SBMs in adults with FC. Reported AEs were diarrhea and abdominal distension [38,39]. An ileal bile acid transporter called elobixibat is a bile acid metabolism modulator [40]. A phase 3 trial showed that SBMs increased significantly compared with placebo. One AE (abdominal pain) was reported in both the elobixibat and the placebo group. Diarrhea was reported in the elobixibat group in 24% of the patients [41]. A drug-free option which is new in the field is the vibrating capsule [42]. A recent phase 3 trial showed that the vibrating capsule was superior to placebo in treating adults with FC, and adverse events were reported on a low base. No one withdrew from the trial. Until now, no studies on children using these modalities have been available [33,36,37,38,40,42,43]. These options could fulfill a place in the future treatment options for FC in children [44]. An overview of detailed information can be found in Table 2.

## 4. Neurostimulation

Neurostimulation in the patient population with FC includes sacral nerve stimulation (SNS) and posterior tibial nerve stimulation (PTNS). For SNS, an electrode is placed in the third sacral foramen, together with the insertion of a stimulation device under the skin near the iliac wings. Currently, understanding of the working mechanism remains unclear.

SNS targets the sacral nerves, which play a crucial role in the regulation of colonic motility and anorectal function. By delivering electrical impulses, SNS aims to restore normal bowel function and alleviate symptoms associated with FC. Studies suggest that SNS may enhance neural communication within the enteric nervous system, leading to improved defecation patterns [9]. For the treatment of FI, SNS is already accepted, but that is not yet the case for the treatment of FC [45]. In 2012, a small retrospective study, for the first time, included thirteen patients (all girls, age 10–18 years) with functional constipation according to the ROME III criteria not responding to intensive oral and rectal laxative treatment, who were assigned for sacral neuromodulation [35]. At presentation, none of the patients had spontaneous defecation or felt the urge to defecate and all patients had severe abdominal pain. After sacral nerve modulation (SNM), 11 (of 12) had a normal spontaneous defecation pattern of ≥2 times a week without medication, felt the urge to defecate, and perceived less abdominal pain without relapse of symptoms until 6 months after implantation. Another study from the same group investigated the long-term efficacy of sacral neuromodulation (SNM) in children and adolescents aged 10–20 years with chronic functional constipation refractory to conservative treatments. Similarly, SNS provided benefits that sustained over a prolonged period of time [46]. Subsequently, others also reported similar results of SNS in children with intractable chronic constipation, fecal an/or urinary incontinence, or urinary retention [47]. An American study, in 22 patients (55% male, median 12 years) with refractory constipation severe enough to require an antegrade continence enema (ACE), reported that the median ACE frequency decreased from seven per week at baseline to one per week at 12 months (*p* < 0.0001). Ten children (45%) had their cecostomy/appendicostomy closed. Six (27%) children experienced complications after SNS that required further surgery [48].

A recent open-label, pragmatic RCT included 67 patients (14–80 years) with idiopathic slow transit constipation (STC), characterized by a defecation frequency of fewer than three times per week and unresponsiveness to conservative treatments [49]. Patients were randomized to receive either SNM or personal conservative treatment (PCT). Patients allocated to PCT continued care as usual (i.e., conservative nonoperative treatment) under the guidance of their referring physician and equal to their treatment before study participation. After 6 months, 22 (53.7%) patients were successfully treated with SNM versus 1 (3.8%) patient with PCT (odds ratio 36.4, 95% CI 3.4-387.5, *p* = 0.003). Patients in the SNM group experienced an increase in bowel movements per week, along with improvements in quality-of-life measures. The incidence of adverse events, including wound infection and the need for repositioning of the device due to a lack of response or lead replacement, was comparable between the two groups [50,51,52,53,54,55]. In addition, the cost-effectiveness analysis suggested that SNM may be a cost-effective option for patients with refractory idiopathic STC [56].

A key aspect of the study design was the exclusion of patients with outlet obstruction, including both anatomical disorders such as internal prolapse and functional outlet obstruction, where patients are unable to relax pelvic floor muscles during defecation. This exclusion was critical to ensure that the study population consisted solely of patients whose constipation was due to impaired colonic motility rather than obstructive defecation, which would probably not respond to SNM.

Due to smaller body size and reduced subcutaneous fat in pediatric patients, placing the neurostimulator can be challenging, even with the advent of smaller implants in recent years [36]. This difficulty may result in discomfort from the device and potentially require repositioning or removal. As children grow, the stimulation lead might shift, leading to decreased effectiveness, the need for device reprogramming, or additional revision surgeries. Additionally, the psychological effects of living with an implanted device should not be overlooked. Nevertheless, the overall morbidity and impact of sacral nerve stimulation (SNM) are generally considered lower than those associated with surgical alternatives such as antegrade continence enemas, (subtotal) colonic resection or stoma formation.

Another option is posterior tibial nerve stimulation (PTNS), a method using a transcutaneous or percutaneous option, i.e., TPTNS and PPTNS. This approach is similar to SNS. It indirectly stimulates the same sacral nerve roots. Its use in adults has been studied, but in children, the literature is sparse [57]. In 2023, a study assessed the efficacy and safety of TPTNS in children aged 4–14 years with FC [58]. Twenty children completed the study. There was a significant increase in the amount of SBM prior to TPTNS. In addition, episodes of FI were significantly decreased. Nineteen of the included children would recommend this therapy for the treatment of FC. Another uncontrolled study reported on children, 8–12 years of age, with refractory functional constipation fulfilling the Rome IV criteria receiving TPTNS [59]. Of the 36 enrolled patients, 28 children completed the study, receiving TPTNS for 4 weeks. Sixteen patients (57.1%) extended the intervention period for 4 extra weeks, receiving 8 weeks of intervention. TPTNS led to significant improvements in stool consistency, frequency of defecation, and bowel function scores, with a reduction in abdominal pain.

TPTNS is a safe, non-invasive method which might be used as a treatment option in children with therapy-resistant FC.

## 5. Transanal Irrigation

Transanal irrigation (TAI), also known as retrograde irrigation, is a technique used to cleanse the colon by flushing it with fluid through a catheter or cone device inserted via the anus. Among the various colorectal patient populations experiencing FI, children with FC have shown the most favorable response to TAI [60]. Studies report an average success rate of 62% [61], indicating that TAI is a safe and effective treatment option for this group.

The most commonly reported side effect is mild pain during the irrigation, affecting approximately 20% of patients [44]. However, this discomfort rarely results in discontinuation of therapy [61]. One of the key limitations of TAI is the need for ongoing assistance, particularly in younger children. In one study, only 2 out of 26 (8%) children with FC, 6–18 years of age at start of TAI, were able to perform TAI independently, highlighting the importance of discussing the procedure’s time demands and dependence on caregivers with families prior to initiation of this treatment [62].

Despite these challenges, parental satisfaction with TAI remains high. One study reported that 88% of parents were satisfied with TAI [63]. Health-related Quality of life (HRQoL) assessments using the PedsQL and PedsQL-GI instruments yielded scores of 79 and 74, which are respectively high compared to other studies in similar patient populations [64,65].

Overall, TAI represents a valuable therapeutic option for children with FC, particularly when conventional educational and medical interventions fail, with or without FI.

## 6. Surgical Interventions

The primary indication for surgical intervention in patients with FC is the failure of medical management, which includes laxatives and rectal treatments such as enemas and transanal irrigation with continuation of symptoms related to constipation and/or FI. Other indications for surgical intervention are persistent FI and significant rectosigmoid dilation [66,67,68,69,70]. A variety of surgical options have been documented for the management of therapy-resistant FC. These include intrasphincteric botulinum toxic injections, antegrade continence enemas (ACEs), resection of the colon (sigmoid resection) with colorectal or coloanal anastomosis, total proctocolectomy with an ileoanal anastomosis, and a diverting stoma [71]. To date, there is scarce evidence for surgical treatment of childhood constipation, and surgical strategies differ widely among physicians [72].

## 7. Botulinum Toxin

Botulinum toxin (BTX) injection into the sphincter complex has traditionally been used in children with HD, internal anal sphincter achalasia, or anal fissures. However, its use has expanded to include children with therapy-resistant FC, particularly in cases with evidence of severe withholding behavior or anal sphincter dysfunction [66,67]. The mechanism of action involves temporary local muscle relaxation by reducing sphincter contraction. These sphincter problems are ideally documented by anorectal manometry, but often treatment for the sphincters is given empirically [73,74]. Typically, BTX (100 units) is diluted with saline (2 cc) and administered via multiple small injections circumferentially around the sphincter complex, often distributed across the four quadrants.

This procedure is considered safe, with a review of 1332 injections reporting adverse effects in fewer than 1% of cases, all of which were self-limiting [75]. Retrospective studies have reported a response rate of approximately 70% in children with FC [76]. However, a more recent prospective study comparing children with FC and those with HD found a lower response rate of 47% in the FC group versus 76% in the Hirschsprung group [77]. It is important to note that BTX has traditionally been used to relax the internal anal sphincter in patients with HD. In contrast, patients with FC typically have a normal recto-anal inhibitory reflex, suggesting that internal sphincter dysfunction is not responsible for their defecation difficulties. Another retrospective multicenter study assessing the effect of botulinum toxin in children with FC found a response rate of 21%. Despite this, provider and parental reported success were significantly higher [76]. However, a more recent prospective study comparing children with FC and those with Hirschsprung disease found a lower response rate of 47% in the FC group versus 76% in the Hirschsprung group [77]. Another retrospective multicenter study assessing the effect of botulinum toxin in children with FC found a response rate of 21%. Despite this, provider and parental reported success were significantly higher [78]. A recent randomized study also found that both intrasphincteric botulinum toxin injection and intrarectal botulinum toxin with electromotive drug administration (BoNTA/EMDA) significantly improved constipation symptoms and stool form within one month. BoNTA/EMDA may offer additional advantages, as it is less costly, associated with fewer comorbidities, and can be performed without general anesthesia [79].

Given this, botulinum toxin injection remains a valuable therapeutic option for managing therapy-resistant FC in children; however, several important considerations must be taken into account. Firstly, administration in children generally requires anesthesia, which adds risk and complexity to the intervention. Additionally, the long-term efficacy of botulinum toxin injections remains uncertain, as there is limited data on how many injections are necessary, how long the effects last, and how patients respond over time. The financial burden of this treatment is also significant, often making it inaccessible for some families. To better define its role in pediatric FC management, a placebo-controlled, long-term follow-up trial is urgently needed to establish appropriate dosing, injection frequency, and sustained clinical benefit [78,80].

## 8. Antegrade Colonic Enemas

The use of antegrade colonic enemas (ACEs) has emerged as an effective therapeutic option for children with therapy-resistant FC. ACE procedures are generally considered minimally invasive and have demonstrated favorable clinical outcomes in both organic constipation and FC [66]. By enabling antegrade administration of enemas, this approach facilitates regular colonic evacuation, thereby preventing fecal impaction and reducing episodes of FI. Various enema solutions can be used, including saline and PEG [81].

Since the technique was first introduced by Malone et al. in 1990 [82], several surgical modifications have been developed, with a recent shift toward less invasive options such as laparoscopic percutaneous cecostomy and the Malone appendicostomy [83,84]. Reported success rates for ACE therapy vary significantly across studies, ranging from 32% to 100%. In approximately 15% of cases, ACE treatment was discontinued due to clinical improvement, while in 8%, it was halted due to failure, necessitating more invasive surgical intervention [85]. One of the ways to predict the success of ACE is to try transanal irrigation first. A stepped approach to the treatment of bowel dysfunction, suggesting the use of TAI before ACE treatment, was proposed by Mosiello et al. [86]. The importance of this stepped approach is emphasized by a retrospective cohort study involving 38 patients with FC, assessing outcomes of ACE surgery [87]. In that study, 100% of those who had successful rectal enema management before ACE continued to have success after ACE. In another retrospective study involving 62 children who were treated with ACE for constipation with or without FI, 94% used TAI before ACE [88].

Complication rates also vary widely, from 6% to 100%, with another surgical intervention required in 0% to 34% of cases [85]. An analysis comparing the effect of a cecostomy and a Malone appendicostomy in children with FC, found higher rates of both minor and major complications following laparoscopic percutaneous cecostomy, with 35% of patients requiring re-intervention due to major complications [89]. Common complications include skin lacerations, stoma stenosis, granulation tissue formation, leakage of enema solution, and tube dislodgement [85].

Despite these risks, ACE treatment is generally well-regarded by patients and their families and has been associated with sustained improvements in bowel habits and gastrointestinal HRQoL [68,69,90]. Ideally, colonic motility is assessed when a patient has therapy-resistant constipation. Based on these results, therapy can be determined, and ACE is almost always helpful, particularly in patients with slow but consistent motility (high amplitude propagated contractions (HAPCs)) throughout the colon [81]. Even for patients with segmental dysmotility, ACE only was effective in more than 90% of patients. This treatment has led to a dramatic reduction in the use of colon resections for severe FC [81]. Also, there is good evidence to support the fact that consistent antegrade emptying of the colon improves the colonic function over time.

## 9. Resection and Ostomies

The literature on (partial) colonic resections in children with FC remains limited. Surgical decision-making is typically guided by findings from contrast enemas and colonic manometry [91]. Therapy-resistant FC in children can lead to the development of a megarectum, megasigmoid, or both [91]. In such cases, rectosigmoid resection has been shown to be effective, as removal of the dilated and dysfunctional bowel segment often results in improved stooling patterns, disappearance of FI, and enhanced QoL. An ACE for antegrade flushes following such a resection is usually beneficial [92].

Levitt et al. reported successful outcomes in 12 of 15 children with therapy-resistant FC and FI who underwent transanal rectosigmoid resection with primary coloanal anastomosis [93]. This operation involved preservation of the anal canal just like for a Hirschsprung’s pull-through, but as opposed to a sigmoid resection includes removal of the rectum. However, this study showed that some patients had FI after the surgical treatment of their constipation (likely from loss of the rectal reservoir), which led to the recommendation of a laparoscopic low anterior resection [92] (with preservation of the rectum), and with better results, avoiding fecal incontinence seen in the transanal rectosigmoid group. More recently, that same group found that antegrade flushes alone (with no resection) helped the majority of patients, even those with a dysmotile sigmoid, leading to almost no patients needing a surgical resection of the colon [81]. Therefore, it can be concluded from this new data that the vast majority of patients do not need any colon segment resected.

Additionally, a prospective study found that 64% of patients who failed to respond to cecostomy or stoma achieved significant clinical improvement following segmental or total colonic resection. Notably, resection allowed 75% of children with a preexisting stoma to return to anal defecation. It is important to note that this group of patients who failed antegrade flushes and then went on to sigmoid resection, in retrospect, were those with pelvic floor dyssynergia or anal sphincter dysfunction. Therefore, botulinum toxin treatments of the pelvic floor and anal sphincters and/or pelvic floor physical therapy should be performed prior to consideration of a surgical resection of the colon [81].

It is important to trouble-shoot the antegrade flush before concluding it has failed. Reflux into the terminal ileum by the flush can occur over time if the ileocecal valve becomes incompetent. A solution for this is to catheterize into the right colon if the patient is performing intermittent catheterization of the Malone, or to extend the length of the tube in the cecostomy [94].

Children with colonic dysmotility or slow-transit constipation may benefit from subtotal or total colectomy, on rare occasions, and only after failure of ACE [67,95]. In a single-center study involving 37 children who underwent sigmoid resection or subtotal colectomy for therapy-resistant constipation, 91% of parents reported high postoperative satisfaction [96]. Similarly, a retrospective review of 31 children treated with laparoscopic sigmoid resection combined with a Malone appendicostomy reported that 95% of patients achieved fecal cleanliness with antegrade flushes [92].

These findings highlight the importance of individualized assessment in children with therapy-resistant FC unresponsive to medical management and ACE. Colonic resection should be viewed as a last-resort intervention and should only be considered after thorough evaluation by a multidisciplinary team.

In the most severe and therapy-resistant cases, the creation of a temporary or permanent stoma for bowel diversion may be warranted. Ileostomy is particularly beneficial for younger children with diffuse colonic dysmotility whose symptoms significantly impair growth and development [67]. Even in older children who fail to respond to ACE, temporary diversion can lead to functional improvement and, potentially, successful stoma reversal, especially when guided by repeat manometric evaluation of the diverted colon [97]. In such a case, if the colon remains inert (no HAPCs throughout), and therefore indicates diffuse colonic dysmotility, a colonic resection with ileorectal anastomosis may be necessary.

The risks associated with stoma creation must be considered. In one study, where ileostomy was the predominant type (72.5%) of diversion used, the overall complication rate reached 95%. Of these, 71.1% were complications requiring re-intervention under anesthesia or specialized management. Common complications included ileostomy prolapse [98], abdominal pain (56.5%), difficulty with stool passage (37.1%), leakage (35.5%), and ileus (27.4%). Ultimately, stoma closure was achieved in 52.5% of patients [99].

These data highlight the complexity of managing therapy-resistant FC and reinforce the need for careful multidisciplinary evaluation before pursuing surgical interventions.

Thorough patient selection is crucial. In children and adolescents with functional constipation—after ruling out endocrine, metabolic, neurological, and obstructive causes—behavioral and psychological factors often have a significant influence. It is essential to address these factors, as the vast majority of patients experience symptom resolution without requiring additional pharmacological or surgical treatment.

## 10. Conclusions

FC is a common, difficult, and frequently long-lasting problem in children. In cases of therapy-resistant FC, more treatment options are being developed. Studies assessing new pharmacologic options compared to the current conventional therapies are increasing, but at the moment, research in children is scarce. Large multi-center trials comparing these new pharmacological approaches are necessary to identify the most effective strategies. Future research on new ion exchangers in children will hopefully give us more insight into optimizing treatment options in children with FC. Neurostimulation, especially PTNS, seems to be a good approach, with a low adverse event rate; however, data is scarce. TAI and ACE are valuable options with high patient and parent treatment satisfaction, next to pharmacological options. Surgical options are invasive, and careful decision-making by an experienced multidisciplinary team is necessary.

## Figures and Tables

**Table 1:** Overview of new pharmacologic options in children with functional constipation.

	LUBIPROSTONE	LINACLOTIDE	PRUCALOPRIDE	PLECANATIDE
**MECHANISM OF ACTION**	Activates ClC-2 chloride channels, increasing intestinal fluid secretion and promoting motility	Guanylate cyclase-C agonist; increases cGMP, stimulating chloride and bicarbonate secretion via CFTR channel	Selective 5-HT4 receptor agonist; enhances colonic motility and contractions	Guanylate cyclase-C agonist; increases cGMP, stimulating chloride and bicarbonate secretion via CFTR channel
**TYPICAL DOSAGE** **(ADULTS)**	12 or 24 mcg once daily	145 mcg once daily	2 mg once daily	3 or 6 mg once daily
**MOST COMMON SIDE EFFECT**	Nausea, diarrhea	Diarrhea	Headache, abdominal pain	Diarrhea
**USE IN PEDIATRICS**	Limited data, not advised as treatment option	Limited data	Not advised as treatment option	No published clinical data

**Table 2:** Overview of future study directions for children with functional constipation.

FEATURE	TENAPANOR	MIZAGLIFLOZIN	ELOBIXIBAT	VIBRATING CAPSULE
**MECHANISM OF ACTION**	Inhibits intestinal sodium/hydrogen exchanger 3 (NHE3), reduces sodium absorption, reduces fluid resorption	Sodium-glucose co-transporter 1 (SGLT1) inhibitor; reduces glucose and water absorption	Ileal bile acid transporter (IBAT) inhibitor; increases bile acid delivery to colon to stimulate secretion of fluids and stimulates motility	Provides mechanical stimulation to the colon via vibration to enhance motility
**TYPICAL DOSAGE**	50 mg twice daily	5 or 10 mg once daily	10 mg once daily	Once daily before night time, works after 15 h
**MOST COMMON SIDE EFFECT**	Diarrhea	Diarrhea	Abdominal pain, diarrhea	Mild abdominal discomfort, sensation of vibration
**USE IN PEDIATRICS**	No published clinical data	No published clinical data	No published clinical data	No published clinical data

## Data Availability

No new data were created or analyzed in this study. Data sharing is not applicable to this article.

## References

[1] Koppen I.J.N., Vriesman M.H., Saps M., Rajindrajith S., Shi X., van Etten-Jamaludin F.S., Di Lorenzo C., Benninga M.A., Tabbers M.M. (2018). Prevalence of Functional Defecation Disorders in Children: A Systematic Review and Meta-Analysis. J. Pediatr..

[2] Bloem M.N., Baaleman D.F., Thapar N., Robets S.E., Koppen I.J.N., Benninga M.A. (2025). Prevalence of functional defecation disorders in European children: A systematic review and meta-analysis. J. Pediatr. Gastroenterol. Nutr..

[3] Fedele F., Fioretti M.T., Scarpato E., Martinelli M., Strisciuglio C., Miele E. (2024). The ten “hard” questions in pediatric functional constipation. Ital. J. Pediatr..

[4] AbouZeid A.A., Abdelmalek A.A.R., Hassen O.G., Mohammad S.A. (2024). Overlooked anterior anal misplacement: A ‘forme fruste’ anomaly and potentially correctable cause of constipation. Egypt. Pediatr. Assoc. Gaz..

[5] Keshtgar A.S., Ward H.C., Clayden G.S., Sanei A. (2004). Thickening of the internal anal sphincter in idiopathic constipation in children. Pediatr. Surg. Int..

[6] Mulhem E., Khondoker F., Kandiah S. (2022). Constipation in Children and Adolescents: Evaluation and Treatment. Am. Fam. Physician.

[7] Hyams J.S., Di Lorenzo C., Saps M., Shulman R.J., Staiano A., van Tilburg M. (2016). Functional Disorders: Children and Adolescents. Gastroenterology.

[8] Benninga M.A., Nurko S., Faure C., Hyman P.E., St. James Roberts I., Schechter N.L. (2016). Childhood Functional Gastrointestinal Disorders: Neonate/Toddler. Gastroenterology.

[9] Vriesman M.H., Koppen I.J.N., Camilleri M., Di Lorenzo C., Benninga M.A. (2020). Management of functional constipation in children and adults. Nat. Rev. Gastroenterol. Hepatol..

[10] Gordon M., MacDonald J.K., Parker C.E., Akobeng A.K., Thomas A.G. (2016). Osmotic and stimulant laxatives for the management of childhood constipation. Cochrane Database Syst. Rev..

[11] Tabbers M.M., Di Lorenzo C., Berger M.Y., Faure C., Langendam M.W., Nurko S., Staiano A., Vandenplas Y., Benninga M.A. (2014). Evaluation and treatment of functional constipation in infants and children: Evidence-based recommendations from ESPGHAN and NASPGHAN. J. Pediatr Gastroenterol. Nutr..

[12] Sinopoulou V., Gordon M., Rajindrajith S., Hathagoda W., Rane A.B., Sedghi A., Tabbers M.M., Di Lorenzo C., Saps M., Benninga M.A. (2024). How do we define therapy-resistant constipation in children aged 4-18 years old? A systematic review with meta-narrative synthesis. BMJ Paediatr. Open.

[13] Lacy B.E., Levy L.C. (2008). Lubiprostone: A novel treatment for chronic constipation. Clin. Interv. Aging.

[14] Barish C.F., Drossman D., Johanson J.F., Ueno R. (2010). Efficacy and Safety of Lubiprostone in Patients with Chronic constipation. Dig. Dis. Sci..

[15] Drossman D.A., Chey W.D., Johanson J.F., Fass R., Scott C., Panas R., Ueno R. (2008). Clinical trial: Lubiprostone in patients with constipation-associated irritable bowel syndrome—Results of two randomized, placebo-controlled studies. Aliment. Pharmacol. Ther..

[16] Hussain S.Z., Labrum B., Mareya S., Stripling S., Clifford R. (2021). Safety of Lubiprostone in Pediatric Patients With Functional Constipation. J. Pediatr. Gastroenterol. Nutr..

[17] Benninga M.A., Hussain S.Z., Sood M.R., Nurko S., Hyman P., Clifford R.A., O’Gorman M., Losch-Berindon T., Mareya S., Lichtlen P. (2022). Lubiprostone for Pediatric Functional Constipation: Randomized, Controlled, Double-Blind Study With Long-term Extension. Clin. Gastroenterol. Hepatol..

[18] Gordon M., Grafton-Clark C., Rajindrajith S., Benninga M.A., Sinopoulou V., Akobeng A.K. (2024). Treatments for intractable constipation in childhood. Cochrane Database Syst. Rev..

[19] Elkaragy E.S., Shamseya M.M., Metwally R.H., Mansour E.R., Lashen S.A. (2024). Efficacy of lubiprostone for functional constipation treatment in adolescents and children: Randomized controlled trial. J. Pediatr. Gastroenterol. Nutr..

[20] Bryant A.P., Busby R.W., Bartolini W.P., Cordero E.A., Hannig G., Kessler M.M., Pierce C.M., Solinga R.M., Tobin J.V., Mahajan-Miklos M. (2010). Linaclotide is a potent and selective guanylate cyclase C agonist that elicits pharmacological effects locally in the gastrointestinal tract. Life Sci..

[21] Serra J., Pohl D., Azpiroz F., Chiarioni G., Ducrotté P., Gourcerol G., Hungin A.P.S., Layer P., Mendive J.M., Pfeifer J. (2020). European society of neurogastroenterology and motility guidelines on functional constipation in adults. Neurogastroenterol Motil.

[22] Baaleman D.F., Gupta S., Benninga M.A., Bali N., Vaz K.H., Yacob D., Di Lorenzo C., Lu P.L. (2021). The Use of Linaclotide in Children with Functional Constipation or Irritable Bowel Syndrome: A Retrospective Chart Review. Paediatr. Drugs.

[23] Di Lorenzo C., Khlevner J., Rodrigez-Araujo G., Xie W., Huh S.Y., Ando M., Hyams J.S., Nurko S., Benninga M.A., Simon M. (2024). Efficacy and safety of linaclotide in treating functional constipation in paediatric patients: A randomised, double blind, placebo-controlled, multicentre, phase 3 trial. Lancet Gastroenterol Hepatol.

[24] (2020). Highlights of Prescribing Information, Prucalopride.

[25] Diederen K., Mugie S.M., Benninga M.A. (2015). Efficacy and safety of prucalopride in adults and children with chronic constipation. Expert Opin. Pharmacother..

[26] Briejer M.R., Bosmans J.P., Van Daele P., Jurzak M., Heylen L., Leysen J.E., Prins N.H., Schuurkers J.A. (2001). The in vitro pharmacological profile of prucalopride, a novel enterokinetic compound. Eur. J. Pharmacol..

[27] Sajid M.S., Hebbar M., Baig M.K., Li A., Philipose Z. (2016). Use of Prucalopride for Chronic Constipation: A Systematic Review and Meta-analysis of Published Randomized, Controlled Trials. J. Neurogastroenterol. Motil..

[28] Quigley E.M., Vandeplasscshe L., Kerstens R., Ausma J. (2009). Clinical trial: The efficacy, impact on quality of life, and safety and tolerability of prucalopride in severe chronic constipation—A 12-week, randomized, double-blind, placebo-controlled study. Aliment Pharmacol. Ther..

[29] Mugie S.M., Korczowski B., Bodi P., Green A., Kerstens R., Ausma J., Ruth M., Levine A., Benninga M.A. (2014). Prucalopride Is No More Effective Than Placebo for Children with Functional Constipation. Gastroenterology.

[30] Velez A., Kaul A., El-Chammas K.I., Knowlton L., Madis E., Sahay R., Fei L., Stiehl S., Santucci N.R. (2024). Safety and Effectiveness of Prucalopride in Children with Functional Constipation with and without Upper Symptoms. Paediatr. Drugs.

[31] Nurko S., Saps M. (2014). Treating constipation with prucalopride: One size does not fit all. Gastroenterology.

[32] Love B.L. (2019). Plecanatide for Treatment of Chronic Constipation and Irritable Bowel Syndrome. Am. J. Med..

[33] Chang L., Chey W.D., Imdad A., Almario C.V., Bharucha A.E., Diem S., Greer K.B., Hanson B., Harris L.A., Ko C. (2023). American Gastroenterological Association-American College of Gastroenterology Clinical Practice Guideline: Pharmacological Management of Chronic Idiopathic Constipation. Gastroenterology.

[34] Cash B.D., Sharma A., Walker A., Laitman A.P., Chang L. (2023). Plecanatide for the treatment of chronic idiopathic constipation and irritable bowel syndrome with constipation: Post hoc analyses of placebo-controlled trials in adults with severe constipation. Neurogastroenterol. Motil..

[35] Miner P.B., Koltun W.D., Wiener G.J., De La Portilla M., Prieto B., Shailubhai K., Layton M.B., Barrow L., Magnus L., Griffin P.H. (2017). A Randomized Phase III Clinical Trial of Plecanatide, a Uroguanylin Analog, in Patients With Chronic Idiopathic Constipation. Am. J. Gastroenterol..

[36] Lembo A.J., Friedenberg K.A., Fogel R.P., Edelstein S., Zhao S., Yang Y., Rosenbaum D.P., Chey W.D. (2023). Long-term safety of tenapanor in patients with irritable bowel syndrome with constipation in the T3MPO-3 study. Neurogastroenterol. Motil..

[37] Chey W.D., Lembo A.J., Yang Y., Rosenbaum D.P. (2021). Efficacy of Tenapanor in Treating Patients With Irritable Bowel Syndrome With Constipation: A 26-Week, Placebo-Controlled Phase 3 Trial (T3MPO-2). Am. J. Gastroenterol..

[38] Fukudo S., Endo Y., Hongo M., Nakajima A., Abe T., Kobayashi H., Nakata T., Nakajima T., Sameshima K., Kaku K. (2018). Safety and efficacy of the sodium-glucose cotransporter 1 inhibitor mizagliflozin for functional constipation: A randomised, placebo-controlled, double-blind phase 2 trial. Lancet Gastroenterol. Hepatol..

[39] Inoue T., Takemura M., Fushimi N., Fujimori Y., Onozato T., Kurooka T., Asari T., Takeda H., Kobayashi M., Nishibe H. (2017). Mizagliflozin, a novel selective SGLT1 inhibitor, exhibits potential in the amelioration of chronic constipation. Eur. J. Pharmacol..

[40] Luthra P., Camilleri M., Burr N.E., Quigley E.M.M., Black C.J., Ford A.C. (2019). Efficacy of drugs in chronic idiopathic constipation: A systematic review and network meta-analysis. Lancet Gastroenterol. Hepatol..

[41] Nakajima A., Seki M., Taniguchi S., Ohta A., Gillberg P.G., Mattsson J.P., Camilleri M. (2018). Safety and efficacy of elobixibat for chronic constipation: Results from a randomised, double-blind, placebo-controlled, phase 3 trial and an open-label, single-arm, phase 3 trial. Lancet Gastroenterol Hepatol..

[42] Rao S.S.C., Quigley E.M.M., Chey W.D., Sharma A., Lembo A.J. (2023). Randomized Placebo-Controlled Phase 3 Trial of Vibrating Capsule for Chronic Constipation. Gastroenterology.

[43] King A.J., Chang L., Li Q., Liu L., Zhu Y., Pasricha P.J., Wang J., Siegel M., Caldwell J.S., Edelstein S. (2024). NHE3 inhibitor tenapanor maintains intestinal barrier function, decreases visceral hypersensitivity, and attenuates TRPV1 signaling in colonic sensory neurons. Am. J. Physiol. Gastrointest. Liver. Physiol..

[44] van der Zande J.M.J., Koppen I.J.N., Di Lorenzo C., Lu P.L., Benninga M.A. (2025). Pharmacological treatment for children with constipation: Present and future. Expert Opin Pharmacother.

[45] Thaha M.A., Abukar A.A., Thin N.N., Ramsanahie A., Knowles C.H. (2015). Sacral nerve stimulation for faecal incontinence and constipation in adults. Cochrane Database Syst. Rev..

[46] van der Wilt A.A., van Wunnik B.P., Sturkenboom R., Han-Geurts I.J., Melenhorst J., Benninga M.A., Baeten C.G., Breukink S.O. (2016). Sacral neuromodulation in children and adolescents with chronic constipation refractory to conservative treatment. Int. J. Colorectal. Dis..

[47] Sulkowski J.P., Nacion K.M., Deans K.J., Minneci P.C., Levitt M.A., Mousa H.M., Alpert S.A., Teich S. (2015). Sacral nerve stimulation: A promising therapy for fecal and urinary incontinence and constipation in children. J. Pediatr. Surg..

[48] Lu P.L., Asti L., Lodwick D.L., Nacion K.M., Deans K.J., Minneci P.C., Teich S., Alpert S.A., Yacob D., Di Lorenzo C. (2017). Sacral nerve stimulation allows for decreased antegrade continence enema use in children with severe constipation. J. Pediatr. Surg..

[49] Heemskerk S.C.M., Dirksen C.D., van Kuijk S.M.J., Benninga M.A., Baeten C.I.M., Masclee A.A.M., Melenhorst J., Breukink S.O. (2024). Sacral Neuromodulation Versus Conservative Treatment for Refractory Idiopathic Slow-transit Constipation: The Randomized Clinical No.2-Trial. Ann. Surg..

[50] Lu P.L., Koppen I.J.N., Orsagh-Yentis D.K., Leonhart K., Ambeba E.J., Deans K.J., Minneci P.C., Teich S., Diefenbach K.A., Alpert S.A. (2018). Sacral nerve stimulation for constipation and fecal incontinence in children: Long-term outcomes, patient benefit, and parent satisfaction. Neurogastroenterol. Motil..

[51] Park C.K., Wang L., Koppen I.J.N., Alpert S.A., Diefenbach K.A., Wood R.J., Bali N., Vaz K., Yacob D., Di Lorenzo C. (2024). Sacral nerve stimulation leads to long- term improvement in fecal incontinence and quality of life for children with functional and organic defecation disorders. Neurogastroenterol. Motil..

[52] Besendörfer M., Knorr C., Kirchgatter A., Müller H., Reis Wolfertstetter P., Matzel K.E., Diez S. (2024). Sacral neuromodulation in children and adolescents with defecation disorders. Neurogastroenterol. Motil..

[53] Vriesman M.H., Wang L., Park C., Diefenbach K.A., Levitt M.A., Wood R.J., Alpert S.A., Benninga M.A., Vaz K., Yacob D. (2020). Comparison of antegrade continence enema treatment and sacral nerve stimulation for children with severe functional constipation and fecal incontinence. Neurogastroenterol. Motil..

[54] Dudding T.C. (2012). Sacral nerve stimulation for constipation in children: A caution for a treatment in development. Dis. Colon Rectum..

[55] Trinidad S., Jensen A., Holder M., Elsner A., Rosen N., Garrison A., Rymeski B., Frischer J.S. (2023). Sacral Nerve Stimulation in Children with Medically Refractory Fecal Incontinence or Severe Constipation. J. Pediatr Surg..

[56] Heemskerk S.C.M., van der Wilt A.A., Penninx B.M.F., Kleijnen J., Melenhorst J., Dirksen C.D., Breukink S.O. (2024). Effectiveness, safety and cost-effectiveness of sacral neuromodulation for idiopathic slow-transit constipation: A systematic review. Colorectal Dis..

[57] Hamedfar M., Ghaderi F., Salehi Pourmehr H., Soltani A., Ghojazadeh M., Vahed N. (2024). Posterior tibial nerve electrical stimulation in chronic constipation: A systematic review and meta-analysis. Gastroenterol Hepatol. Bed. Bench..

[58] Velasco-Benitez C., Villamarin E., Mendez M., Linero A., Hungria G., Saps M. (2023). Efficacy of transcutaneous posterior tibial nerve stimulation in functional constipation. Eur. J. Pediatr..

[59] Mayara Padilha Rego R., Machado N.C., Carvalho M.A., Graffunder J.S., Fraguas C., Ortolan E.V.P., Lourenção P.L.T.A. (2024). Transcutaneous Posterior Tibial Nerve Stimulation: An Adjuvant Treatment for Intractable Constipation in Children. Biomedicines.

[60] Caruso A.M., Milazzo M.P.M., Bommarito D., Girgenti V., Amato G., Paviglianiti G., Casuccio A., Catalano P., Cimador M., Di Pace M.R. (2021). Advanced Management Protocol of Transanal Irrigation in Order to Improve the Outcome of Pediatric Patients with Fecal Incontinence. Children.

[61] Bolia R., Goel A., Thapar N. (2024). Transanal irrigation in children with functional constipation: A systematic review and meta-analysis. J. Pediatr. Gastroenterol. Nutr..

[62] Baaleman D.F., Wegh C.A.M., Hoogveld M.T.A., Benninga M.A., Koppen I.J.N. (2022). Transanal Irrigation in Children: Treatment Success, Quality of Life, Adherence, Patient Experience, and Independence. J. Pediatr. Gastroenterol. Nutr..

[63] Koppen I.J., Kuizenga-Wessel S., Voogt H.W., Voskeuil M.E., Benninga M.A. (2017). Transanal Irrigation in the Treatment of Children With Intractable Functional Constipation. J. Pediatr. Gastroenterol. Nutr..

[64] Hartman E.E., Pawaskar M., Williams V., McLeod L., Dubois D., Benninga M.A., Joseph A. (2014). Psychometric properties of PedsQL generic core scales for children with functional constipation in the Netherlands. J. Pediatr. Gastroenterol. Nutr..

[65] Varni J.W., Bendo C.B., Nurko S., Shulman R.J., Self M.M., Franciosi J.P., Saps M., Pohl J.F. (2015). Pediatric Quality of Life Inventory (PedsQL) Gastrointestinal Symptoms Module Testing Study Consortium. Health-related quality of life in pediatric patients with functional and organic gastrointestinal diseases. J. Pediatr..

[66] Siminas S., Losty P.D. (2015). Current Surgical Management of Pediatric Idiopathic Constipation: A Systematic Review of Published Studies. Ann. Surg..

[67] Wood R.J., Yacob D., Levitt M.A. (2016). Surgical options for the management of severe functional constipation in children. Curr. Opin. Pediatr..

[68] Baaleman D.F., Vriesman M.H., Lu P.L., Benninga M.A., Levitt M.A., Wood R.J., Yacob D., Di Lorenzo C., Koppen I.J.N. (2023). Long-Term Outcomes of Antegrade Continence Enemas to Treat Constipation and Fecal Incontinence in Children. J. Pediatr. Gastroenterol. Nutr..

[69] Christison-Lagay E.R., Rodriguez L., Kurtz M., St Pierre K., Doody D.P., Goldstein A.M. (2010). Antegrade colonic enemas and intestinal diversion are highly effective in the management of children with intractable constipation. J. Pediatr. Surg..

[70] De la Torre L., Cogley K., Cabrera-Hernández M.A., Frias-Mantilla J.E., Wehrli L.A. (2019). Transanal proximal rectosigmoidectomy. A new operation for severe chronic idiopathic constipation associated with megarectosigmoid. J. Pediatr. Surg..

[71] Deloyers L. (1958). Technic permitting the easy assurance of continuity of the colon & conservation of sphincter after excision of the left transverse hemicolon & entire left colon; possible inclusion of rectum. J. Chir..

[72] Koppen I.J., Kuizenga-Wessel S., Lu P.L., Benninga M.A., Di Lorenzo C., Lane V.A., Levitt M.A., Wood R.J., Yacob D. (2016). Surgical decision-making in the management of children with intractable functional constipation: What are we doing and are we doing it right?. J. Pediatr. Surg..

[73] Tiusaba L., Jacobs S.E., Bokova E., Tyraskis A., Russell T.L., Al-Shamaileh T., Feng C., Teeple E., Darbari A., Levitt M.A. (2003). Functional Constipation Refractory to Medical Management: The Anal Sphincters are the Problem. J. Pediatr. Surg. Open.

[74] Jacobs S.E., Tiusaba L., Bokova E., Russell T.L., Al-Shamaileh T., Feng C., Badillo A.T., Darbari A., Levitt M.A. (2023). Functional constipation refractory to medical management: The colon is the problem. J. Pediatr. Surg..

[75] Halleran D.R., Lu P.L., Ahmad H., Paradiso M.M., Lehmkuhl H., Akers A., Hallagan A., Bali N., Vaz K., Yacob D. (2019). Anal sphincter botulinum toxin injection in children with functional anorectal and colonic disorders: A large institutional study and review of the literature focusing on complications. J. Pediatr. Surg..

[76] Zar-Kessler C., Kuo B., Belkind-Gerson J. (2018). Botulinum toxin injection for childhood constipation is safe and can be effective regardless of anal sphincter dynamics. J. Pediatr. Surg..

[77] Baaleman D.F., Hallagan A., Halleran D.R., Orsagh-Yentis D.K., Levitt M.A., Wood R.J., Benninga M.A., Bali N., Vaz K.H., Yacob D. (2023). Anal Botulinum Toxin in Children with Hirschsprung Disease and Functional Constipation: A Prospective Cohort study. Eur. J. Pediatr. Surg..

[78] Patel D., Saikumar P., Jayaraman M., Desai C., Rosen J., Rodriguez L. (2025). Efficacy of anal botulinum toxin injection in children with functional constipation. J. Pediatr. Gastroenterol. Nutr..

[79] Kajbafzadeh A.M., Sharifi-Rad L., Nabavizadeh B., Ladi-Seyedian S.S., Alijani M., Farahmand F., Motamed F., Alimadadi H., Fallahi A., Fallahi G.H. (2020). Intrarectal Electromotive Botulinum Toxin Type A Administration in Children With Intractable Constipation: A Randomized Clinical Trial. Am. J. Gastroenterol..

[80] de Geus A., Koppen I.J.N., Flint R.B., Benninga M.A., Tabbers M.M. (2023). An Update of Pharmacological Management in Children with Functional Constipation. Paediatr Drugs.

[81] Ahmad H., Smith C., Witte A., Lewis K., Reeder R.W., Garza J., Zobell S., Hoff K., Durham M., Calkins C. (2024). Antegrade Continence Enema Alone for the Management of Functional Constipation and Segmental Colonic Dysmotility (ACE-FC): A Pediatric Colorectal and Pelvic Learning Consortium Study. Eur. J. Pediatr. Surg..

[82] Malone P.S., Ransley G., Kiely E.M. (1990). Preliminary report: The antegrade continence enema. Lancet.

[83] Lawal T.A., Rangel S.J., Bischoff A., Peña A., Levitt M.A. (2011). Laparoscopic-assisted Malone appendicostomy in the management of fecal incontinence in children. J. Laparoendosc. Adv. Surg. Tech. A.

[84] Rodriguez L., Flores A., Gilchrist B.F., Goldstein A.M. (2011). Laparoscopic-assisted percutaneous endoscopic cecostomy in children with defecation disorders (with video). Gastrointest Endosc..

[85] Jonker C.A.L., van der Zande J.M.J., Benninga M.A., de Jong J.R., Di Lorenzo C., Lu P.L., Tabbers M.M., de Vries R., Koppen I.J.N., Gorter R.R. (2025). Antegrade Continence Enemas for Pediatric Functional Constipation: A Systematic Review. J. Pediatr. Surg..

[86] Mosiello G., Marshall D., Rolle U., Crétolle C., Santacruz B.G., Frischer J., Benninga M.A. (2017). Consensus Review of Best Practice of Transanal Irrigation in Children. J. Pediatr. Gastroenterol. Nutr..

[87] Reppucci M.L., Nolan M.M., Cooper E., Wehrli L.A., Schletker J., Ketzer J., Peña A., Bischoff A., De la Torre L. (2022). The success rate of antegrade enemas for the management of idiopathic constipation. Pediatr. Surg. Int..

[88] Jonker C.A.L., Koppen I.J.N., Benninga M.A., de Jong J.R., Gorter R.R. (2025). Outcomes and Complications of Chait Trapdoor Cecostomy in Pediatric Patients with Therapy-Resistant Constipation and Fecal Incontinence: A 14-Year Retrospective Study. Eur. J. Pediatr. Surg..

[89] Halleran D.R., Vilanova-Sanchez A., Rentea R.M., Vriesman M.H., Maloof T., Lu P.L., Onwuka A., Weaver L., Vaz K.K., Yacob D. (2019). A comparison of Malone appendicostomy and cecostomy for antegrade access as adjuncts to a bowel management program for patients with functional constipation or fecal incontinence. J. Pediatr. Surg..

[90] Srinivas S., Halaweish I., Knaus M.E., Ahmad H., Griffin K.L., Stephenson K.G., Yossef L., Trimble C., Jimenez A.L.N., Lu A. (2024). Outcomes of children with constipation and autism spectrum disorder treated with antegrade continence enemas. J. Pediatr. Gastroenterol. Nutr..

[91] Levitt M.A., Mathis K.L., Pemberton J.H. (2011). Surgical treatment for constipation in children and adults. Best Pract. Res. Clin. Gastroenterol..

[92] Gasior A., Reck C., Vilanova-Sanchez A., Diefenbach K.A., Yacob D., Lu P., Vaz K., Di Lorenzo C., Levitt M.A., Wood R.J. (2018). Surgical management of functional constipation: An intermediate report of a new approach using a laparoscopic sigmoid resection combined with malone appendicostomy. J. Pediatr. Surg..

[93] Levitt M.A., Martin C.A., Falcone R.A., Peña A. (2009). Transanal rectosigmoid resection for severe intractable idiopathic constipation. J. Pediatr. Surg..

[94] Pearlstein H., Wang L., Thompson B.P., Wood R.J., Levitt M.A., Bali N., Vaz K., Yacob D., Di Lorenzo C., Lu P.L. (2024). Significance of retrograde flow with antegrade continence enemas in children with fecal incontinence and constipation. J. Pediatr. Gastroenterol. Nutr..

[95] Eradi B., Hamrick M., Bischoff A., Frischer J.S., Helmrath M., Hall J., Peña A., Levitt M.A. (2013). The role of a colon resection in combination with a Malone appendicostomy as part of a bowel management program for the treatment of fecal incontinence. J. Pediatr. Surg..

[96] Kuizenga-Wessel S., Koppen I.J.N., Zwager L.W., Di Lorenzo C., de Jong J.R., Benninga M.A. (2017). Surgical management of children with intractable functional constipation; experience of a single tertiary children’s hospital. Neurogastroenterol. Motil..

[97] Rodriguez L., Colliard K., Nurko S., Flores A., Buchmiller T.L. (2022). Diverting Ileostomy in Children With Functional Constipation: A Study Evaluating the Utility of Colon Manometry. J. Pediatr. Gastroenterol. Nutr..

[98] Sparks E.A., Velazco C.S., Fullerton B.S., Fisher J.G., Khan F.A., Hall A.M., Jaksic T., Rodriguez L., Modi B.P. (2017). Ileostomy Prolapse in Children with Intestinal Dysmotility. Gastroenterol. Res. Pract..

[99] Vriesman M.H., Noor N., Koppen I.J.N., Di Lorenzo C., de Jong J.R., Benninga M.A. (2020). Outcomes after enterostomies in children with and without motility disorders: A description and comparison of postoperative complications. J. Pediatr. Surg..

